# Stunting and Underweight among Adolescent Girls of Indigenous Communities in Telangana, India: A Cross-Sectional Study

**DOI:** 10.3390/nu16050731

**Published:** 2024-03-03

**Authors:** Padmaja Ravula, Kavitha Kasala, Soumitra Pramanik, Aravazhi Selvaraj

**Affiliations:** 1 International Crops Research Institute for the Semi-Arid Tropics (ICRISAT), Hyderabad 502324, India; padmaja.ravula@icrisat.org (P.R.); soumitra.pramanik@icrisat.org (S.P.); aravazhi.selvaraj@worldveg.org (A.S.); 2 World Vegetable Center, International Crops Research Institute for the Semi-Arid Tropics (ICRISAT), Hyderabad 502324, India

**Keywords:** malnutrition, indigenous communities, adolescent girls, stunting, dietary practices, nutrition awareness, policies

## Abstract

India’s indigenous groups remain vulnerable to malnutrition, despite economic progress, reflecting the reliance on traditional agriculture and the problems of poverty and inadequate education and sanitation. This mixed-methods study analyzed the incidence, causes and determinants of chronic malnutrition, measured through stunting, thinness and underweight among adolescent indigenous girls in Telangana. Using 2017 data on 695 girls aged 11–18 years from 2542 households, the analysis showed that 13% had normal nutritional status, while 87% were stunted, underweight or thin. Early adolescents (11–14 years) had higher underweight prevalence (24.4%), while late adolescents (15–18 years) showed greater stunting (30%). Regressions identified key influencing factors. Higher education levels of heads of households and the girls themselves alongside household toilet access significantly improved nutritional status and reduced stunting and underweight. The sociocultural emphasis on starchy staple-based diets and early marriage also impacted outcomes. Tackling this crisis requires mainstreaming nutrition across development agendas via comprehensive policies, education, communication and community participation. Further research can guide context-specific solutions. But, evidence-based investments in indigenous education, livelihoods, sanitation and women’s empowerment are the first steps. Nutrition-sensitive development is indispensable for indigenous groups to fully participate in and benefit from India’s progress.

## 1. Introduction

Malnutrition remains a major challenge for human development in India, despite economic progress. High population levels, socioeconomic disparities and inadequate healthcare access contribute to continued malnutrition prevalence, especially among children and adolescents [1,2,3,4]. As per the World Health Organization (WHO) (the World Health Organization (WHO) is a specialized agency of the United Nations responsible for international public health; it is headquartered in Geneva, Switzerland, and has six regional offices and 150 field offices worldwide), adolescents are persons aged 10–19 years. This transition period to adulthood is critical for health, demographic, social and economic development [5]. There is an insufficient exploration of the dual burden of malnutrition among adolescents in low- and middle-income countries (LMIC) [6]. Within the South Asian context, the prevalence of undernutrition in children and adolescents persists at a significantly high level compared to other regions, despite showing a decline over the past three decades [7,8,9]. However, there is a paucity of studies that have systematically quantified the dual burden of malnutrition among adolescents in India, and this gap extends to other South Asian countries also [10,11,12,13].

However, studies show higher malnutrition among Indian adolescents, especially in rural and tribal areas [14,15,16]. Nutritional assessments of adolescents can thus provide key evidence to formulate targeted strategies and programs.

India has the world’s largest adolescent population at 253 million, comprising 120 million girls and 133 million boys, or 20% of the population [17]. Whereas, tribal groups, officially termed Scheduled Tribes (ST) (the Scheduled Castes and Scheduled Tribes are officially designated groups of people and among the most disadvantaged socio-economic groups in India; the terms are recognized in the Constitution of India and the groups are designated in one or other of the categories (https://en.wikipedia.org/wiki/Scheduled_Castes_and_Scheduled_Tribes, accessed on 20 December 2023), comprise 8.6% (over 104 million) of India’s population and predominantly reside in rural, underserved areas [18].

Several studies demonstrate widespread nutrient deficiency and undernutrition among adolescent girls in rural and tribal areas in India [19,20,21,22,23,24,25,26,27,28,29,30,31,32,33,34,35,36]. Rapid growth in adolescence requires increased nutrient intake monitoring and support to achieve full developmental potential [37]. However, data associating physical development with socioeconomic factors are limited in India. Such data could aid authorities in formulating policies for adolescent wellbeing. Some data exist on the nutritional status of indigenous adolescents from Andhra Pradesh, Gujarat, Karnataka, Kerala, Madhya Pradesh, Maharashtra, Odisha, Tamil Nadu and West Bengal [38,39].

A burdening cycle of malnutrition has been revealed among rural Indian women by data from the National Family Health Survey rounds 3 and 4 (NFHS-3 and NFHS-4) (the National Family Health Survey (NFHS) is a large-scale, multi-round survey conducted with a representative sample of households throughout India; the NFHS-3 was conducted in two phases between November 2005 and August 2006, while NFHS-4 was conducted in 2015–2016 and provides information on population, health and nutrition for India and each state and union territory—for more details please visit http://rchiips.org/nfhs/, accessed on 20 December 2023). This nutritional deficiency appears to stem from a convergence of factors, including poverty, food insecurity, gender inequality, and lack of access to healthcare. The cycle begins with malnourished infant girls, who grow up to be malnourished adolescents. They marry early due to the societal setting, get pregnant at an early age with an ill-fit body—often during adolescence—and thereafter give birth to malnourished children. Social structures of marriage have contributed to such a vicious cycle. As the youngest members of their families and adolescents suddenly upgraded to the status of women, these girls have no capacity or authority to negotiate their nutritional needs or make demands for their children’s (especially if girls) food or healthcare needs. This was more pronounced among the indigenous communities that were examined in this study.

Despite economic growth, undernutrition persists as a major health issue in developing countries [40]. Recent Indian data indicates a 26.7% thinness prevalence and 34.1% stunting among adolescents [11,41]. Studies from Africa show 4.5–29% thinness among adolescent school students, influenced by parental education, family income, dietary diversity, meal frequency and residency [42,43,44,45,46,47]. Unhealthy school food environments and peer pressure over nutritional choices also impact status. Gender disparities exist, with higher stunting among Ethiopian adolescent boys [48]. In South Asia, 11% stunting, 39% thinness and 55% anemia prevalences are reported among adolescent girls [17]. Analyses of Indian data show 27.4% stunting, 24.4% thinness, 4.8% overweight and 1.1% obesity among adolescents. Stunting was higher among girls and older adolescents, while thinness was higher for boys and early adolescents. Overweight was slightly higher for boys and early adolescents. Socioeconomic factors influencing adolescent malnutrition include gender, wealth, social status, religion and maternal education [49]. Over 50% of stunting and thinness is reported for late adolescent girls in Chhattisgarh [50]. Despite constitutional protection, positive policies and earmarked budgets, India’s 104 million tribal people remain among the poorest and most nutritionally deprived social groups (Ministry of Tribal Affairs, GOI, 2016–2017) (the Ministry of Tribal Affairs is the Nodal Ministry for overall policy, planning and the coordination of programs of development for Scheduled Tribes; detailed report available at https://tribal.nic.in/writereaddata/AnnualReport/AnnualReport2016-17.pdf, accessed on 20 December 2023). The discrimination that exists in the social structure across India encompasses gender, caste, class and ethnic identity, which impinges on the development of all disadvantaged groups and particularly on their standard of living, dietary patterns, health and education. Women experience discrimination in terms of diet and access to food as members of a specific caste, class or ethnic group, in addition to facing gendered vulnerabilities; this scenario was similar for occupational groups. Research by Palriwala [51] revealed that despite economic growth and reduced poverty and hunger rates, the decline in malnutrition, especially in women, has been extremely slow; malnutrition in the form of iron deficiency and anemia has in fact increased in certain regions and continues to hold good even now (https://indiafoundation.in/articles-and-commentaries/malnutrition-and-its-foundation-in-the-social-structure-a-study-on-indian-women/, accessed on 20 December 2023). Similarly, about 63% of adolescent boys and 42% of girls were undernourished (<5th BMI age percentiles of National Health and Nutrition Examination Survey) for a selected sample from nine states of India. A significant association between undernutrition and socio-economic parameters like type of family, size of land holding and occupation of head of household was observed [38]. The traditional food systems of indigenous populations provide nourishment and hold social and cultural value for communities [52]; the shift from subsistence to commercial agriculture has resulted in environmental degradation, the loss of traditional agricultural practices and the displacement of local varieties of adapted species [53]. The Global Nutrition Report [54] highlights that indigenous people are more likely to bear the burden of undernutrition and malnutrition, which disproportionately affects women and children [55].

Continuing on this discussion, the study of the indigenous adolescent subpopulation in India is imperative for several reasons rooted in the unique challenges and disparities faced by this specific demographic group. First and foremost, indigenous adolescents represent a distinctive cultural and socio-economic subset characterized by distinct cultural traditions, gender and social norms. Understanding their experiences is essential for crafting targeted interventions and policies that acknowledge and respect their cultural diversity.

Furthermore, the existing literature often lacks comprehensive insights into the nutrition, health, education and socio-economic status of indigenous adolescents in India. By focusing on this subpopulation, this manuscript aims to bridge this knowledge gap and contribute valuable data that can inform evidence-based strategies for improvement.

In summary, India’s economic growth has not adequately translated to improved living conditions for indigenous communities. Vital policy actions include protecting land and resources, increasing agricultural productivity and strengthening regional governance where tribes reside [56].

This background motivated an investigation into malnutrition prevalence among adolescent girls in three tribal-dominated Telangana districts. Stunting and thinness were assessed using anthropometry. Focus group discussions (FGDs) and interviews examined dietary customs influencing nutrition and health.

## 2. Methodology

### 2.1. Study Sites and Participants

**Figure 1:**
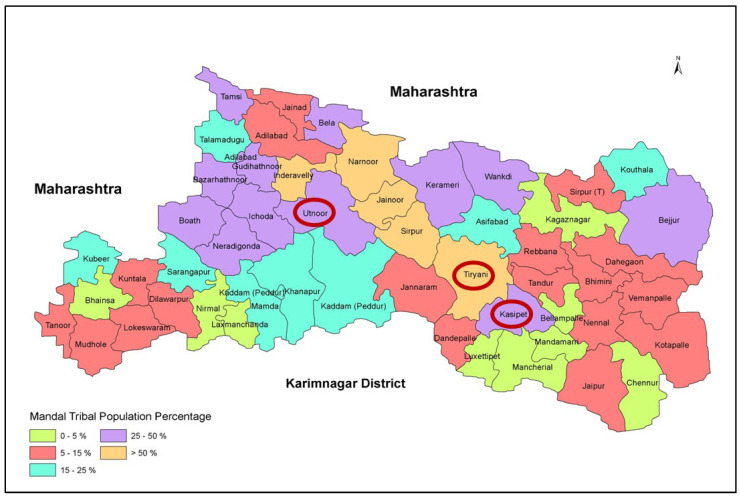
Spatial location of the study areas. Source: ICRISAT Remote Sensing Unit.

The erstwhile district of Adilabad (under the united Andhra Pradesh state) was reorganized and divided into four districts in October 2016: Adilabad, Kumuram Bheem Asifabad, Mancherial and Nirmal. In this study, a cross-sectional survey was conducted in one mandal (a mandal is an administrative division in some parts of India, constituting a subdivision of a district or a subdistrict) from each of the three districts (excluded: Nirmal district), namely, Utnoor (Adilabad district), Tiryani (Kumuram Bheem Asifabad district) and Kasipet (Mancherial district) (Figure 1). As can be seen from the figure, two of the selected mandals had a 25–50% tribal population and one mandal had a more than 50% tribal population.

The erstwhile Adilabad district in India where the present study was conducted is home to various indigenous communities (about 8 tribes), each with its unique composition and distribution. According to the India census 2011, the second most dominant indigenous tribes were the Gond tribe (the people belonging to indigenous communities in India are referred to as Scheduled Tribes in India; https://tribal.nic.in/, accessed on 20 December 2023) and the Lambadas tribe and accounted for about 75% of tribal presence among all other tribes in the region. Similarly, among the 2542 indigenous households surveyed (out of 4648 sample households), the Gonds comprise the largest group, accounting for 55%. The Lambadas, another prominent tribe, made up 14% of the sample households, while the Kolam tribe constituted 10%. Interestingly, the Naikpods, accounting for only 1% in the Adilabad district, had a higher representation of 13% among the indigenous sample households.

**Figure 2:**
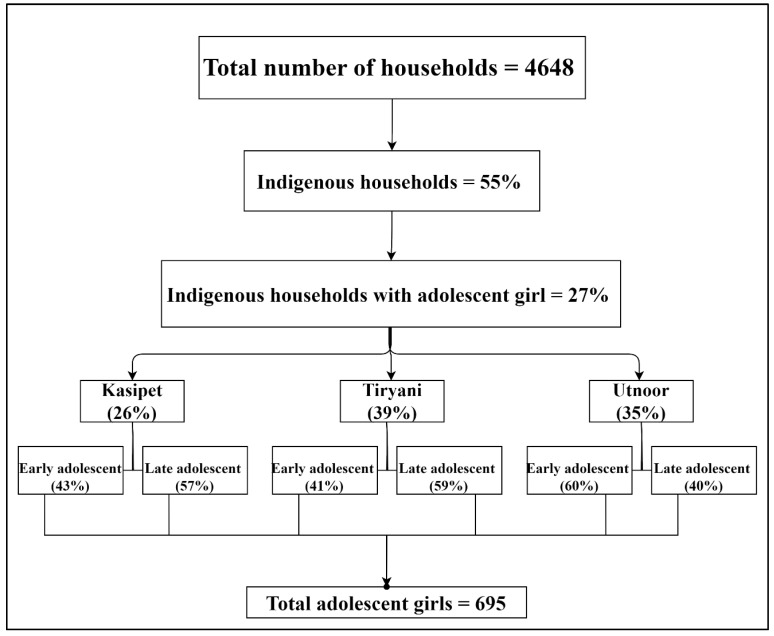
Distribution of sample households, indigenous households and indigenous households with adolescent girl/s in all three locations. Source: Field survey, ICRISAT Nutri-food Basket Project. Note: The percentage values are approximations to the nearest whole number.

The primary data collection was carried out in two phases during the months of May and September 2017 from 2542 indigenous households from the three mandals. Out of 2542 indigenous community households, 55% of them belonged to Gond tribes, and 27% of them specifically had at least one adolescent girl between the ages of 11 and 18 years. The distribution of indigenous households with adolescent girls across the three mandals was as follows: In Kasipet, 26% of the indigenous households had at least one adolescent girl in their household. Moving to Tiryani, this percentage was slightly higher at 39%. Lastly, in Utnoor, 35% of the indigenous households had adolescent girls. Altogether, these three mandals comprised a total of 693 households that had at least one adolescent girl. Therefore, a sample comprising 695 adolescent girls who were available at the time of the survey were selected for the present study. These statistics indicate the prevalence of indigenous households and highlight the specific focus on adolescent girls within this community, which is the focus of the present paper (Figure 2).

More specifically, in Kasipet, 43% of the adolescent girls fell into the early adolescent category (between 11 and 14 years of age), while the remaining 57% belonged to the late adolescent group (above 14–18 years). Similarly, in Tiryani, 41% of the adolescent girls were classified as early adolescents, while 59% were categorized as late adolescents. Utnoor displayed a different pattern, with 60% of the adolescent girls falling into the early adolescent age group and the remaining 40% belonging to the late adolescent category. One plausible explanation for this is that the majority of the adolescent girls attended schools in Utnoor as a day scholar. Utnoor mandal has many schools located in different clusters within the mandal. In Kasipet and Tiryani mandals, due to limited schools for the adolescent age group, there is more enrolment in hostels by adolescent girls. Hence, in Kasipet and Tiryani mandals, there was a lower percentage of the early adolescent sample in this study. Upon completion of high school, the adolescent girls return back to their villages and, therefore, we see more numbers of late adolescents in these two mandals.

### 2.2. Data and Methods

A mixed-methods approach was adopted for collecting and analyzing the data from the field sites. Quantitative data were gathered through a survey protocol developed using digital tools. The survey protocol was collaboratively devised with input from both the International Crops Research Institute for the Semi-Arid Tropics (ICRISAT) and the Telangana Government National Tribal Health Mission (NTHM). The approved baseline survey protocols were subsequently converted into a tablet-compatible questionnaire using CS Pro software, Version 7.0.2 (U.S Census Bureau). In order to facilitate comprehension, all questions, survey instruments, and informed consent materials were translated into the local language, Telugu. Though questionnaire-based interviews were conducted in the local language, the data collector recorded responses in English on the Android device. The survey protocol covered questions related to demography, livelihoods, incomes, nutrition and assets, among others.

The sample size of the adolescent girls involved in this quantitative survey was drawn from 38 villages that has 171 Integrated Child Development Services (ICDS) centers locally known as “Anganwadi”. Participants were selected through purposive sampling. The identified households were approached during field visits, and the study protocol was verbally explained in the local language (Telugu or Gond). Parents were then sought for and obtained informed consent.

The qualitative study aimed at understanding common dietary patterns and perceptions of food amongst the adolescent girls (11–18 years) and was implemented only in selected villages in the Tiryani mandal of the Kumuram Bheem district. Focus group discussions (FGDs) with adolescent girls were conducted in 4 villages, namely, Kannepalli, Goyagaon, Morriguda and Chinnaredipalli villages in the Tiryani mandal.

The height and weight of the participants were measured using standard tools. Measurements were taken using an anthropometric rod (SECA) for height, recorded to the nearest 0.1 cm, and a portable weighing balance (SECA) for weight, measured to the nearest 0.1 kg.

In July 2017, focus group discussions (FGDs) were conducted, comprising adolescent girls aged 11 to 18 who were non-pregnant and non-lactating. The group sizes varied from eight to eleven participants, and discussions spanned 30 to 55 min. A total of 32 adolescents participated in the FGDs. Each focus group consisted of homogenous members, representing female adolescents from the same indigenous community. Purposive sampling was employed for two distinct groups of the population, with specific features across the selected villages. An FGD checklist was prepared and a questionnaire for conducting key informant interviews with the Anganwadi teachers and workers was developed and internally reviewed.

The trained field investigators facilitated the focus group discussions (FGDs) using an open-ended semi-structured discussion guide. They were assisted by other research support staff of the team. The question checklist, created under the guidance of the principal investigator, served as a guiding tool. It was designed to encompass a variety of nutrition, health, and gender issues, addressing specific questions and concerns raised during the qualitative inquiry. Permission (consent) from each participant was obtained before audio recording all FGDs.

Two coordinators from ICRISAT monitored and validated data collected by the trained enumerators on a periodic basis and attended field-based data collection work intermittently.

Data from each village were systematically arranged and securely transmitted to ICRISAT Headquarters (HQ) via a protected internet server, where they were stored securely. Research staff from ICRISAT conducted visits to the data collection sites for monitoring purposes.

Participants’ Body Mass Index (BMI) was determined based on their height and weight measurements.

The World Health Organization (WHO) criteria were utilized to evaluate malnutrition, encompassing stunting (defined as height-for-age z-scores less than 2 standard deviations below the WHO reference median), thinness (defined as BMI below the 5th percentile), and overweight-obese (defined as BMI above the 85th percentile for age according to the National Health and Nutrition Examination Survey I [NHANES I]). references [57,58]. Anthropometric measures were summarized as means with standard deviations.

Multivariate logistic regression analysis was used to assess the determinants of stunting and underweight for adolescent girls. Two separate models were used and the outcome variables (exposure variables) were considered as follows: model 1 compared stunted girls with non-stunted and model 2 compared underweight adolescents versus adolescents with normal BMI. Explanatory variables, such as adolescent age group, family size, age of the household head, education level of the household head, gender of the household head, per-capita monthly income, availability of a toilet, and education level of the adolescents, were considered as covariates. The models were tested to ascertain the roles played by these variables.

The FGDs were recorded using an audio recorder, which was then transcribed into a text format and validated. Thematic analysis of the qualitative data was carried out to bring about the key findings of the study.

## 3. Results

### 3.1. Socio-Demographic Characteristics

Table 1 displays the socio-demographic status of the participants. Around 65% of the adolescents resided in households where decision-making involved both an adult male and female, followed by households with only a male adult (25%) and those with only a female adult (10%). Over 86% of adolescents belonged to households headed by males. Approximately 62% of household heads had limited education, being either illiterate or able to sign only, while 24% had received secondary education or higher. The average per capita income per month was approximately INR 1338 (approximately USD 20). Notably, around 53% of households had a per capita income less than INR 1000 (approximately USD 15), and approximately 11% had a monthly per capita income exceeding INR 2000 (approximately USD 30). This suggests that a majority of households lived on less than USD 1 per day, placing them below the poverty line. The average family size was 4.68 persons, with 50% of families considered large (having more than five members in total).

Housing conditions were poor and almost all of them lacked access to piped water to homes. Untreated water from tube wells or open-dug wells served as the primary source of drinking water for households. A considerable portion of the houses (81%) lacked proper toilet facilities, resulting in widespread open defecation in the nearby surroundings and adjacent farms.

**Figure 3:**
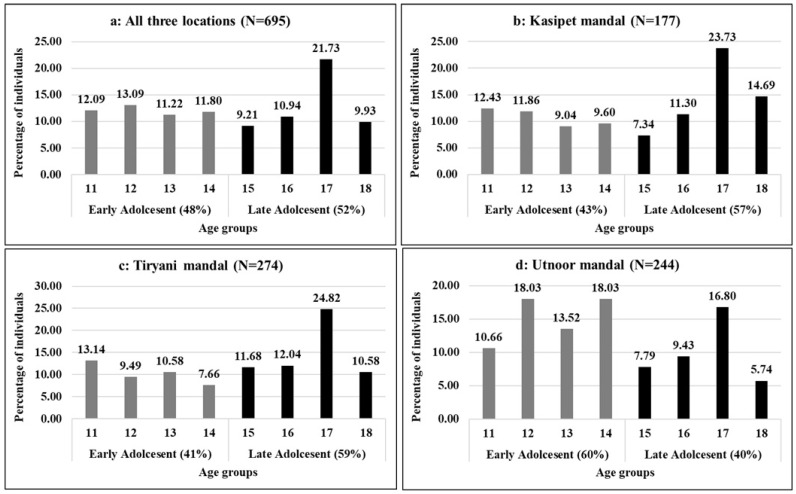
Age-wise distribution of adolescent girls (in %). Source: Field survey, ICRISAT Nutri-food Basket Project.

The age-wise distribution of the sample of adolescent girls (Figure 3) showed variations and provided valuable insights into the distribution of the adolescents across the mandals (Figure 3a–d). In total, 48% of the sample was in the early adolescent category. However, village-wise variations were observed, as can be seen in (Figure 3a).

For instance, in the 18-year age group (3b), Kasipet mandal exhibited a higher percentage of girls compared to the other two mandals. Tiryani mandal (3c) had a greater percentage of girls in the 13-year age group than the other two mandals. Utnoor mandal (3d) showed a higher percentage of girls in the 17-year age group compared to the other two mandals. These variations were also noted in terms of nutritional status.

### 3.2. Nutritional Status

Table 2 provides the mean values of height, weight, and BMI of adolescents based on WHO standard recommendations, as derived from anthropometry data. Significantly (*p* < 0.001), both mean weight and height increased with age. Moreover, a significant (*p* < 0.001) increase in mean BMI was observed for both early and late adolescent girls. Late adolescent girls demonstrated normal BMIs (greater than 18.5 kg/m^2^) compared to their counterparts in the early adolescent years.

**Figure 4:**
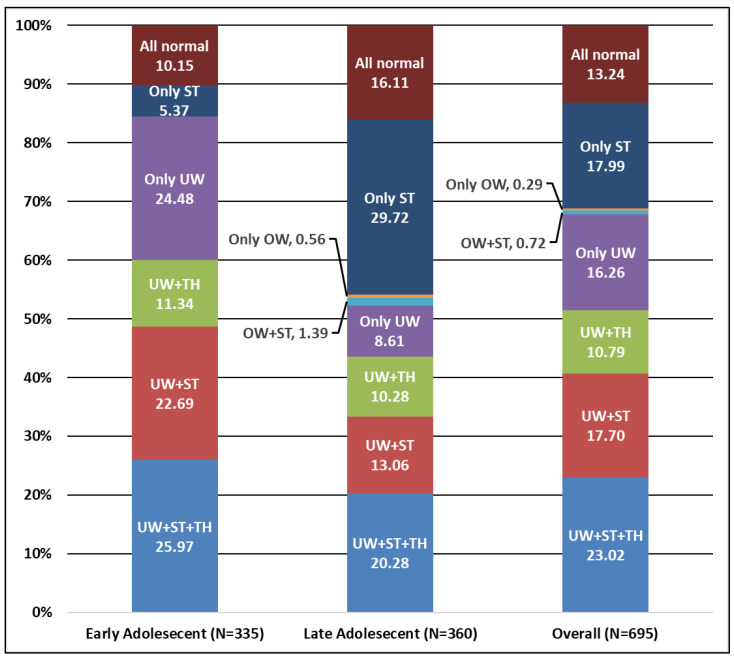
Prevalence of nutritional status indicators among early and late adolescents (in %). Source: Field survey, ICRISAT Nutri-food Basket Project. Note: UW = underweight, OW = overweight, ST = stunting and TH = thinness.

The age-specific nutritional status of the adolescents is presented in Figure 4. This study aimed to assess the nutritional status of early and late adolescents by examining the prevalence of underweight, stunting, thinness and overweight within the two age groups of adolescent girls in the study regions. The findings reveal important differences in nutritional status between the two age groups. The overall prevalence of underweight, stunting and thinness among the participants was found to be 23%. Early adolescents exhibited a slightly higher prevalence of these conditions (about 26%) compared to their late adolescent counterparts (20%). When considering underweight and stunting together, the prevalence decreased to 17.70% overall, with early adolescents displaying a higher prevalence (22.69%) compared to late adolescents (13.06%). Furthermore, the prevalence of underweight and thinness combined was found to be 10.79% overall, with no substantial variation observed between the two age groups.

However, the prevalence of only underweight participants differed significantly, with early adolescents showing a higher prevalence (24.48%) compared to late adolescents (8.61%). Additionally, the prevalence of only stunting differed considerably between the two age groups, with early adolescents displaying a lower prevalence (5.37%) compared to late adolescents (29.72%). Lastly, 13.24% of the overall sample had normal nutritional status, with early adolescents (10.15%) showing a slightly lower prevalence than late adolescents (16.11%). Also, it is to be noted that the prevalence of overweight was not a problem among the sample of adolescent girls and there were no participants classified as solely overweight in the early adolescent group, while a small proportion (0.56%) of late adolescents fell into this category. Similar trends were founds in the case of NFHS-4 and studies from other regions of India [11,59,60]. Therefore, addressing the identified variations in prevalence rates can aid in the development of targeted interventions and policies to improve the overall nutritional well-being of this population.

### 3.3. Determinants of Malnutrition—A Statistical Analysis

Table 3 presents the results of two logistic regression models analyzing determinants of stunting and underweight status among adolescent tribal girls. The logistic regression analysis in Model 1 examines determinants of stunting among adolescent tribal girls, using stunting as the exposure variable compared to non-stunted girls. Multiple variables demonstrated significant associations in relation to stunted growth. For instance, having a late adolescent girl in the household led to a 67.6% higher chance of stunting compared to having an early adolescent girl. In addition, the level of education held by the head of the household played a role, with each higher level of education resulting in a 22% decrease in the odds of stunting (OR = 0.782, *p* = 0.016). Furthermore, access to a toilet in the household appeared to have a positive impact as it was associated with a 31.1% decrease in the odds of stunting (OR = 0.689, *p* = 0.072). Additionally, the education level of the girl herself was also linked to stunting, with each increase in education level resulting in a 20.4% reduction in the odds (OR = 0.796, *p* = 0.077). In the second model, we examined the use of underweight as our exposure variable and discovered that only two variables had significant associations. Surprisingly, late adolescence was linked to a significant 79% decrease in the odds of being underweight (OR = 0.210, *p* < 0.001) when compared to early adolescence. Furthermore, we found that a higher level of education for the head of the household was also significantly associated with a 21.2% decrease in the odds of a girl being underweight (OR = 0.788, *p* = 0.036). The model’s fitness measures, including large LR chi-square values, *p*-values < 0.05 and pseudo R-squared values, clearly demonstrate the strong fit of both models in analyzing the determinants of stunting and underweight. Our findings reveal that late adolescence, lower education of the household head, lack of access to toilets and lower education of girls are significant risk factors for stunting, while late adolescence and higher education of the household head serve as protective factors against underweight among this indigenous population of girls. Some Previous research has also found significant relationships between undernutrition and social factors like family structure, land ownership, and household income source [38].

### 3.4. Qualitative Insights

An analysis of focus group discussion transcripts revealed several key findings related to the dietary habits, health and gender experiences of the vulnerable groups of the indigenous communities. These findings can be grouped into broad categories and include (1) common dietary habits/practices, (2) perceptions of the community’s food culture and the ways in which this culture influences their health, (3) perceived barriers to managing good dietary and sanitary practices, and (4) the effect of Integrated Child Development Services (ICDS), especially Anganwadi, on the diets and nutritional well-being of the vulnerable target beneficiaries. These findings are elaborated below.

In all the study regions, members of various social groups included non-vegetarian foods in their diets. During the focus group discussions, participants were prompted to share insights into their favorite foods, the significance of these choices, the frequency of consumption, and how they managed their food intake while balancing work and home responsibilities. Nearly all participants reported consuming 2 to 3 meals daily, with diets primarily revolving around rice (cereals) accompanied by vegetables or pulses. Roti made from sorghum and pearl millet was a common element in meals across the selected villages, except in one village where rice was the predominant cereal. The discussions on special foods provided to adolescents, such as jaggery and dry coconut, highlighted the impact of personal circumstances and economic levels on their dietary patterns.

The FGDs on perceptions about the importance of different food groups revealed their understanding about nutrition and health. In this respect, we conducted two separate focus group discussions (FGDs) with two groups of adolescent girls—early and late adolescents (Table 4). In the discussion with the early adolescent category, 10% of the participants considered energy-rich foods as important for nutrition, while a higher percentage of 45% prioritized foods rich in micronutrients. Additionally, 45% of early adolescents recognized the significance of protein-rich foods. Moving to the late adolescent category, 20% emphasized the importance of energy-rich foods; only 20% of late adolescents emphasized the importance of foods rich in micronutrients, reflecting a relatively lower awareness of their nutritional value, whereas 60% of late adolescents recognized the significance of protein-rich foods, which was significantly more than in the early adolescent group. Table 4 reflects the perceptions of adolescents about the significance of different food groups, which were definitely different from their actual consumption. Here, the adolescents perceived that protein-rich foods group were important but they did not actually consume them, which was reflected in their nutritional outcome (stunting). Similar outcomes were affirmed in an exploratory qualitative study conducted by [61], which delved into the lived experiences and perspectives of school adolescents regarding the prevention, causes, and consequences of a double burden of malnutrition in Debre Berhan City, Ethiopia. The study revealed that a majority of school-aged teenagers lacked a comprehensive understanding of the effects and preventive measures associated with the double burden of malnutrition, showcasing variations in knowledge, attitude, experience, and perspectives. This observation aligns with the findings reported in other studies addressing the risks and challenges of health behavior [62,63]. For India, such literature is scant and there is no relevant available literature linking the perceptions of food and actual consumption and nutrition outcomes, especially for adolescents in India [29,64]. These findings highlight the varying perceptions and priorities regarding different food groups among adolescents of different age groups and partially substantiate the findings from Figure 4.

**Figure 5:**
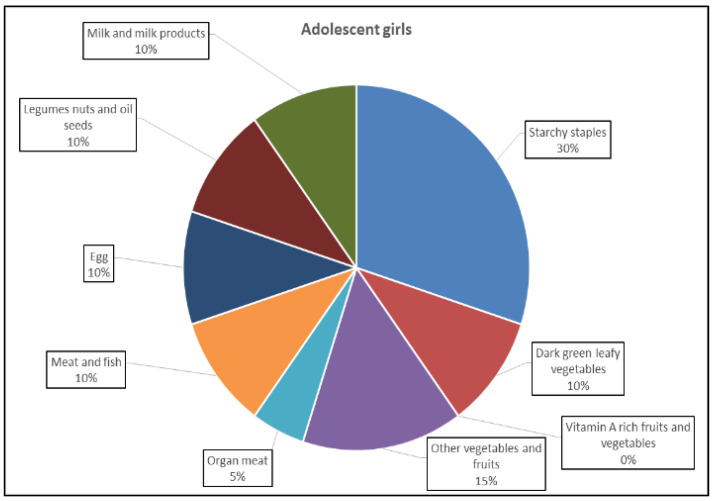
Significance of food groups as perceived by the respondents. Source: Based on qualitative survey, ICRISAT.

To understand the perceptions of the participants of healthy foods, a small exercise was conducted. The group was given twenty seeds, each seed equivalent to a 5% contribution. The group discussed the task among themselves and gave a certain number of seeds for each food group. Based on this exercise, the following perceptions emerged: meat, fish and organ meat and eggs were perceived to be healthy foods; vitamin A-rich fruits and vegetables were not perceived to be the most important foods among the adolescents; the adolescent girls seemed to have a fair idea about the significance of food groups such as dark green leafy vegetables, carrot, meat and eggs that are nutrient-rich but could not specify the nutrients nor their functions. This may be due to a lack of awareness of nutrition (Figure 5).

Fasting was practiced as soon as the girls reached adolescence and puberty. The majority of the adolescent girls in the study region fasted for at least two days in a week. Fasting was observed as the socio-cultural norms in the region warrant this as a way of devoting oneself to religious deities and protecting oneself from evil forces like witchcraft and black magic, among others. During the fasting days, the adolescent girls skip one cooked meal; instead, they have fruits, milk and tea.

In each group, participants reiterated the necessity of traveling to either IB Tandur or Bellampally (nearby villages), emphasizing these markets as the closest sources for acquiring food items. Participants underscored that the extended distance from their habitation to the market was associated with reduced mobility for adolescent girls. The majority of participants clarified that their market visits primarily revolved around acquiring vegetables, oil, and various fried snacks for their households.

### 3.5. Socio-Cultural Norms around Dietary Decisions and Behaviours

Significant dietary changes were observed, particularly concerning the staple food, transitioning from sorghum or millet roti to rice. This shift primarily resulted from a change in agricultural practices, with a move from millet production to rice cultivation. Additionally, the influence of the Targeted Public Distribution System (PDS) of the Government, providing 4 kg of rice per household member each month, partially contributed to this dietary transformation. Eating food outside the household—either cooked food or packaged foods—was not common in the villages a decade ago; the preferences of the adolescents and young children have changed over time and they engage in outside eating behaviors. There has been an increase in petty food vendors and shops stocking packaged foods (especially small packs) in the tribal villages also.

Across the FGDs, a common sentiment among participants was the acknowledgment of the positive aspect of having toilets. Despite this consensus, a limited number of households had toilet facilities situated within their premises.

### 3.6. Government Programs for Adolescent Girls

The adolescent girls were provided with midday meals by the school and no additional food or referral services were provided by the ICDS workers. About thirty percent of Anganwadis did not maintain a list of adolescent girls as there were no programs aimed at them. The participants responded that anemia among pregnant, lactating, child and adolescent girls roughly ranged from five to thirty percent in their respective villages. Some participants denied knowing the percentage of the prevalence of anemia. Adolescent girls were not extensively covered under the ICDS, the Kishori Balika Shakti Yojana scheme. Intermittent supplementary nutrition and very rare nutrition and sanitation awareness campaigns were carried out for adolescent girls.

## 4. Discussion

These findings align with existing literature and findings from various studies. According to a USAID report, the global prevalence of stunting among adolescent girls is 45%, and among boys, it is 20%. The National Institute of Nutrition’s study on adolescents in India indicates that food and nutrient intakes were below the Recommended Dietary Allowance (RDA) compared to their rural counterparts, with a higher deficit in micronutrients such as iron, vitamin A, riboflavin, and free folic acid. The prevalence of undernutrition was relatively higher among adolescents from tribal communities compared to rural counterparts from other social groups. Additionally, the well-known and accepted fact of a higher prevalence of undernutrition among girls is consistent across various Indian communities [38,65,66,67].

The regression analysis also showed lower stunting but higher underweight likelihood among early adolescent girls. Factors like household toilet access and education play key roles in reducing stunting, aligning with past research on socioeconomic influences on undernutrition [38,68,69,70,71,72].

The qualitative insights that this study highlights were also observed by other researchers.

As indicated by Rao et al., [38], a noteworthy correlation exists between undernutrition and socio-economic factors such as family type, landholding size, and the occupation of the household head. Various socio-cultural, economic, and environmental factors play a role in shaping both food intake and health-seeking behaviors [73].

The findings highlight adolescents’ perceptions regarding healthy foods, indicating a positive view towards meat, fish, organ meat, and eggs. These items were commonly recognized as health-promoting. In contrast, vitamin A-rich fruits and vegetables were not accorded the same level of importance in the participants’ perceptions of healthy foods. This suggests a potential gap in awareness or preference for certain nutritious food sources among adolescents.

The majority of animal food sources such as meat and organ meat were perceived to be healthy compared to plant-based food sources. Indigenous communities rely on a wide range of animal food sources, a widely documented consumption practice. Some consumption practices have been adding to the recent debates on sustainable food systems generally. As observed in the results, the diets mainly comprised cereals and pulses cultivated locally. Sorghum and pearl millet roti were integral components of the daily diet of the indigenous communities studied, alongside rice.

Significant dietary shifts, particularly concerning the staple food, were observed, transitioning from sorghum or millet roti to rice. This change in dietary patterns primarily stemmed from a shift in agricultural practices, specifically from millet to rice production. Additionally, the influence of the Government’s Targeted Public Distribution System (PDS), providing a substantial monthly rice allocation per household member, played a notable role in driving this dietary transformation. These shifts underscore the interconnectedness of agricultural practices and government initiatives in shaping dietary habits within the community [74]. Eating food outside the household—either cooked food or packaged foods—was not common in the villages a decade ago; the preferences of adolescents and young children have changed over time and they engage in outside eating behaviors. There has been an increase in petty food vendors and shops stocking packaged foods (especially small packs) in the selected villages as well. As per the perceptions of the participants, cereals and millets provided the major percentage of energy that is important for the health of an individual. Adequate fruit and vegetable consumption remains rare across social groups due to limited availability and accessibility.

Even with governmental provisions, sanitation and hygiene facilities and practices, particularly among the households of adolescents in these communities, remain rudimentary. Enhancing awareness and fostering improved behaviors in this regard can substantially contribute to advancing overall health outcomes. A coordinated, goal-oriented approach with multi-sectoral stakeholders is imperative to further enhance the nutrition literacy and the overarching nutritional outcomes of adolescents and communities. The critical physical and cognitive growth phase of adolescence, for both boys and girls, warrants targeted interventions given the high malnutrition prevalence. These study findings will assist policymakers and program managers in shaping healthcare and developmental schemes for indigenous groups, spotlighting adolescents.

While this study analyzed various factors influencing adolescent nutritional status, potential confounders like physical activity and food types were not examined. Another limitation is the exclusive focus on girls. Additionally, causal conclusions are restricted due to the cross-sectional methodology. Longitudinal data would better reveal the socio-demographic impacts on nutritional status. However, regional studies on indigenous groups examining these relationships are limited. Further in-depth research, especially among 11–18-year-old girls, is recommended. Therefore, comprehensive policies, programs, education drives and behavior change campaigns focused on adolescent girls’ nutrition are strongly recommended for indigenous groups based on this study. Mainstreaming nutrition across developmental agendas is vital alongside decentralized services tackling access barriers. Community participation and leadership must complement top-down efforts.

## 5. Conclusions

Indigenous communities are prone to high malnutrition, particularly undernutrition, due to geographical isolation, food supply uncertainty, inadequate healthcare and sociocultural diet and hygiene norms. This study confirms the vulnerability of adolescent indigenous girls—economically, educationally and nutrition-wise—as evidenced by anthropometric measurements. Beyond the current education programs, there is a crucial need for targeted nutrition literature and behavior change campaigns tailored specifically for rural and indigenous adolescents. These initiatives are essential to address the unique challenges and contribute to improving the overall well-being of these communities.

Underweight, wasting and stunting are unfortunately common among adolescent girls, as multiple studies highlight. A higher stunting prevalence points to the chronicity of malnutrition, perpetuating inter-generational impacts. This constitutes an urgent gendered crisis necessitating customized interventions.

While the conclusions provide directional guidance, more expansive ethnographic and epidemiological studies across diverse geographies can illuminate nuances. Quantifying health budgets, system capacities and policy gaps can also inform structural reform priorities. Impact evaluations of existing schemes on nutritional indicators are needed to showcase replicable best practices. Concurrent action alongside future research is, however, warranted by the scale of malnutrition-linked human and economic costs.

## Figures and Tables

**Table 1:** Household Socio-demographic Features of the Adolescent Girls (N = 693).

Particulars	Values
Household Type (%)	
Male and female adult	65.47
Female adult only	9.93
Male adult only	24.60
Gender of Household Head (%)	
Male	86.47
Female	13.53
Average age of household head (years)	43.00
Education Status of Household Head (%)	
Illiterate or just able to sign	62.30
Up to primary level	13.53
Secondary level or above	24.17
Household Income	
Average monthly per-capita income (in INR)	1338
Income (per-capita/month) ≤ INR 1000 (% of households)	53.38
Income (per-capita/month) between INR 1001–2000 (% of households)	35.83
Income (per-capita/month) > INR 2000 (% of households)	10.79
Family Size	
Average family size (Number of individuals)	4.68
Family size ≤ 4 (% of households)	50.50
Family size 5–6 (% of households)	38.56
Family size > 6 (% of households)	10.94
Sanitation—Toilet Availability (%)	
Yes	18.85
No	81.15

Source: Field survey, ICRISAT Nutri-food Basket Project.

**Table 2:** Mean anthropometric measurements of adolescent girls in Utnoor, Tiryani and Kasipet mandals, Telangana.

Indicator	Early Adolescent	Late Adolescent	Overall
Height (cm)	141.89 (8.96)	150.45 * (5.80)	146.32 (8.62)
Weight (kg)	33.05 (7.42)	42.10 * (7.11)	37.74 (8.55)
BMI (kg/m^2^)	16.22 (2.43)	18.54 * (2.72)	17.42 (2.83)

Source: Field survey, ICRISAT Nutri-food Basket Project. Note: (i) Values within parentheses indicate standard deviations (SDs); (ii) * denotes significant differences between late adolescents and early adolescents.

**Table 3:** Determinants of stunting and underweight of tribal adolescent girls: a logistic regression analysis.

Variable	Model 1	Model 2
	(Exposure Variable: Stunted = 1 vs. Non-Stunted = 0)	(Exposure Variable: Underweight = 1 vs. Normal BMI = 0)
	Odds Ratio (OR)	Odds Ratio (OR)
Adolescent dummy (Early adolescent = 0, Late adolescent = 1)	1.676 *** (0.002)	0.210 *** (0.000)
Household size	1.008 (0.883)	1.067 (0.305)
Head gender (Male = 1, Female = 0)	1.231 (0.396)	1.194 (0.522)
Age of household head (Years)	0.985 (0.129)	0.992 (0.501)
Education of head (Illiterate or just able to sign = 1, Up to primary = 2, Secondary and above = 3)	0.782 *** (0.016)	0.788 ** (0.036)
Monthly per-capita income (In INR “000”)	1.000 (0.553)	1.000 (0.149)
Access to toilet (Available = 1, Otherwise = 0)	0.689 * (0.072)	0.879 (0.570)
Education of adolescent (Illiterate or just able to sign = 1, Up to primary (Grade 1 to 5) = 2, Secondary level and above (Grade 6 and above) = 3	0.796 * (0.077)	0.934 (0.632)
Constant	5.021 *** (0.008)	9.665 *** (0.001)
LR chi^2^ (8)	24.52	92.55
Prob > chi^2^	0.0019	0.0000
Log likelihood	−457.055	−382.598
Pseudo R^2^	0.026	0.108
Number of observations	695	695

Source: Field survey, ICRISAT Nutri-food Basket Project. Notes: (i) *p*-values in parentheses; (ii) ***, ** and * represent 1%, 5% and 10% level of significance, respectively.

**Table 4:** Perceived percentage of significance of food groups (in %).

Food Groups	Early Adolescent	Late Adolescent
Energy-rich	10	20
Micronutrient-rich	45	20
Protein-rich	45	60

Source: Field survey, ICRISAT Nutri-food Basket Project. Note: The information was collected through focus group discussions.

## Data Availability

Data will be made available upon request. The data are not publicly available due to confidentiality concerns.
